# Effect of the Lactation Phases on the Amplitude of Variation in Blood Serum Steroid Hormones and Some Hematochemical Analytes in Three Dairy Cow Breeds

**DOI:** 10.3390/ani14223336

**Published:** 2024-11-20

**Authors:** Esterina Fazio, Arianna Bionda, George Attard, Pietro Medica, Deborah La Fauci, Annalisa Amato, Luigi Liotta, Vincenzo Lopreiato

**Affiliations:** 1 Department of Veterinary Sciences, Messina University, Viale Palatucci 13, 98168 Messina, Italy; pmedica@unime.it (P.M.); deborah.lafauci@unime.it (D.L.F.); annalisa.amato@unime.it (A.A.); luigi.liotta@unime.it (L.L.); vincenzo.lopreiato@unime.it (V.L.); 2 Department of Agricultural and Environmental Sciences—Production, Landscape, Agroenergy, Milan University, Via Celoria 2, 20133 Milan, Italy; arianna.bionda@unimi.it; 3 Department of Rural Sciences and Food Systems, University of Malta, 2080 Msida, Malta; george.attard@um.edu.mt

**Keywords:** analytes, dairy cows, lactation, steroid hormones

## Abstract

Lactation in dairy cows implies comprehensive endocrine and metabolic changes including a systemic electrolytic reaction. Previous studies have rarely considered these specific demands due to the influence of lactation periods. Therefore, this study aimed to assess the effects of early, middle, and late lactation phases on the dynamic changes in serum concentrations of progesterone (P4), 17β-oestradiol (E2), cortisol, and some electrolytes (Ca^++^, Mg^++^, Na^+^, K^+^, Cl^−^, Pi) and biochemical parameters (alanine aminotransferase (ALT), aspartate aminotransferase (AST), lactate dehydrogenase (LDH), creatine kinase (CK), total bilirubin, urea, and iron (Fe^++^) in 10 Holstein, 10 Brown Swiss, and 10 Modicana multiparous healthy dairy cows (4.2 ± 1.7 years of age) sampled at 60-day intervals throughout lactation. Lactation induced significant changes in the concentrations of P4, which peaked at >120–180 days, decreased at >240–300 days, and increased again after 300 days. Cortisol showed an opposite trend to P4, with concentrations progressively decreasing, except for the phase between >240 and 300 days, and a steep drop at >300 days compared to previous phases. Na^+^ concentrations showed the lowest values at 0–60 d and the highest ones at >180–240 days, whereas Mg^++^ showed the highest values at >60–120 d and the lowest at >300 d. Significant correlations were found between P4 with cortisol, Cl^−^ and K^+^, and cortisol with Ca^++^ and LDH. Significant differences in average concentrations of AST, ALT, LDH, Ca^++^, Mg^++^, and Fe^++^ were observed among different dairy cow breeds. Understanding the dynamic changes in hormone levels, electrolytes, and biochemical parameters during different lactation phases, while considering breed differences in dairy cows, is crucial for improving herd health management and milk production in commercial dairy farms.

## Simple Summary

The priority of the lactating animal is to provide the mammary gland with nutrients via metabolic changes orchestrated by several mechanisms, and more specifically, by the dynamic crosstalk between steroid hormones and some hematological analytes. The present study provides new evidence that occurs at the onset of lactation and throughout the lactation period in 10 Holstein, 10 Brown Swiss, and 10 Modicana healthy dairy cows, showing that lactation induces significant changes in circulating progesterone, cortisol, sodium, and magnesium concentrations. The breeds showed a significant effect on liver enzyme (AST, ALT, and LDH) activities and the concentration of calcium and magnesium, according to the early, middle, and late phases of lactation. Significant correlations were recorded for progesterone with cortisol, chlorine, and potassium, and for cortisol with calcium and LDH. Understanding the differences among breeds might improve their management, nutrition, and productivity in commercial dairy farms.

## 1. Introduction

A dairy cow’s adaptive response to physiologically demanding events often involves the mobilization of body tissue reserves, hepatic responses, and mechanisms that ensure electrolyte balance. In an attempt to maintain homeostasis and health, these metabolic processes are in a constant state of flux in response to changes occurring during the various physiological phases throughout the life cycle of the cow. The monitoring of fluctuations in the endocrine secretions and metabolites can provide valuable insight into how cows adapt to these complex events [1,2].

It has been reported that, at parturition, the plasma progesterone (P4) concentration declines, while that of estradiol and cortisol peaks [3,4]. P4 is involved in mammogenesis and the onset of lactation [4,5]. This sudden surge in milk production following parturition is most likely due to the rapid decline of serum P4 concentrations. Lactating dairy cows have subnormal P4 concentrations as a result of a higher blood flow through the liver, which increases the rate of P4 catabolism [6].

Both estrogens and P4 are reported to modulate iron (Fe^++^) status in cyclic and pregnant non-ruminant animals [7], and recently, the existence of an estrogen-iron axis in mares was supported by Satué et al. [8,9,10]. Estrogens increase Fe^++^ release from hepatocytes, enterocytes, and macrophages [11], whereas P4 is reported to have the opposite effect [12].

Stress induces an increased release of adrenocorticotropic pituitary hormone and cortisol, both of which are reported to suppress reproductive function [13,14]. Additionally, while changes in steroid metabolism during lactation have also been associated with reduced fertility [6], glucocorticoids are indispensable for lactogenesis, parturitions, and the maturation of different organs [15,16]. Information on metabolic stress associated with the physiological transformation during the different lactation phases in dairy cows is scarce, while the metabolic and health problems that occur around calving are well-documented [17].

Blood metabolites and mineral profiles of cows during the transition period [18,19] and the lactation phase [2,20] have already been reported in previous studies. However, our review of the literature clearly indicates that previous studies rarely take into account the effects of the different lactation phases on the dynamics of endocrine and hematochemical parameters.

Holstein Friesian, Brown Swiss, and Modicana are dairy breeds selected for different purposes and display different productivity and metabolic responses during the lactation period. Understanding the differences among breeds might improve their management, nutrition, and productivity in commercial dairy farms.

In view of the above, this study used two highly productive cosmopolitan breeds and a local Sicilian breed to support the hypothesis that endocrine and some hematochemical modulations could occur along the early, middle, and late lactation phases, with possible differences between the different breeds.

This study was supported by funds from the BIOTRAK project (Grant number 08SR1091000150-CUP G69J18001000007), which had, as a main focus, the innovation and technological transfer of agro-industrial waste and by-products for use as animal feed with a view toward the circular economy and sustainability of the livestock supply chain. The blood samples collected throughout this project unveiled several interesting pieces of data of physiological relevance, some of which have already been published [21,22]. This current study also forms part of this physiological research cohort. Thus, the objective of this study was to evaluate the effect of lactation phases on the amplitude of flux changes in serum concentrations of progesterone (P4), 17β-oestradiol (E2), cortisol, electrolytes (Ca^++^, Mg^++^, Na^+^, K^+^, Cl^−^, and Pi), and biochemical parameters, such as alanine aminotransferase (ALT), aspartate aminotransferase (AST), lactate dehydrogenase (LDH), creatine kinase (CK), total bilirubin, urea and iron (Fe^++^) in 10 Holstein, 10 Brown Swiss, and 10 Modicana dairy cows.

## 2. Materials and Methods

### 2.1. Animal Housing and Management

The present study was conducted on a commercial farm located within the suburbs of the city of Ragusa (36°53′47″ N, 14°42′24.8″ E), in Sicily, Italy. The herd consisted of 100 milking cows of different breeds, Italian Holstein Friesian, Italian Brown Swiss, and Modicana, and managed in line with the local traditional semi-intensive farming system. Cows were kept in a free stall on a straw-deep litter system. Calves are weaned from dams within 6 h after birth. All the animals selected for this study were kept under the same environmental and managerial protocols.

A group of 30 multiparous healthy dairy cows consisting of 10 Holstein, 10 Brown Swiss, and 10 Modicana were randomly selected from the herd to form the study group. At the time of the first sampling, these selected animals were homogeneous for age (4.2 ± 1.7 years), body condition score (BCS), evaluated using a 1-to-5 scale (2.7 ± 0.3) [23], and stage of lactation (35 ± 20 d). Cows were milked twice daily at 04:30 and 16:30 CEST (Central European Summer Time) and had different average milk production statuses comparable to their particular breed: 28.8 ± 3.3 kg/head/d for Holstein, 27.3 ± 3.1 kg/head/day for Brown Swiss, and 15.0 ± 2.0 kg/head/d for Modicana.

The same feed was given throughout the entire trial period. Meadow hay was delivered ad libitum indoors and in the pasture plots, whereas concentrate was offered twice daily at 08:00 and 14:00 CEST, with the exception of sampling days, when the morning ration was distributed following the completion of sampling. The concentrate was formulated with the inclusion of 8% of dried and pitted olive cakes following an approved EU disciplinary called “QS Sicilia” aimed at the recovery and reuse of agro-industrial by-products. The ingredients and chemical composition of hay and concentrate are reported in Table S1. Cows were given access to pasture in spring and autumn (for a minimum of 6 h during daylight, from 08:00 to 14:00 CEST) but not in summer. Animals had ad libitum access to drinking water, both indoors and outside. The indoor housing was in a free-stall style barn equipped with fans and sprinklers that automatically switch on during hot events to maintain a comfortable temperature. Table S2 reports the mean temperature (T) and relative humidity (RH) recorded daily in Ragusa and the related temperature humidity index (THI), measured using the formula THI = (1.8 × T + 32) − [(0.55 − 0.55 × RH) × (1.8 × T − 26.8)] (National Research Council (U.S.). Committee on Physiological Effects of Environmental Factors on Animals, 1971) [24].

All animals were certified as healthy based on (1) their normal cyclicity activity during the previous breeding seasons, (2) the absence of reproductive pathologies, and (3) the absence of inflammatory and infectious processes that occurred and were treated with antibiotics or anti-inflammatory agents within a month prior to the first blood sampling and throughout the whole experimental period.

### 2.2. Blood Samples

At the start of the study, all selected cows were at the early lactation stage and registered as pregnant at 132 ± 50 d of lactation. This wide range is related to the fact that the animals were confirmed as pregnant on different days of lactation. Following insemination, pregnancy was determined following an ultrasound examination on day 30 of gestation.

Blood samples were collected at 60-day (d) intervals, with day 0 being the calving date. Thus, the periods considered were 0–60 d, 61–120 d, 121–180 d, 181–240 d, 241–300 d, and >300 d following calving and the onset of lactation. Blood was withdrawn through venipuncture of the jugular vein into 10 mL tubes containing a clot activator and separating gel (Terumo Corporation, Tokyo, Japan). Blood samples were centrifuged for 10 min at 2000× *g*; the supernatant serum was collected and stored at −20 °C until further analyses. To minimize the effect of the circadian rhythm on the hormonal measurements, all samples were taken by the same operator in the morning between 7:00 and 9:00 while maintaining the environment as calm as possible.

The experimental protocol was approved by the Ethical Committee of the Department of Veterinary Science of Messina University (code 041/2020). The research complied with the guidelines of Good Clinical Practices and the Italian and European regulations on animal welfare (Directive 2010/63/EU).

### 2.3. Hormonal and Analytic Analyses

Serum E2, P4, and cortisol concentrations were assayed using a homologous solid-phase two-site chemiluminescent immunometric assay (Immulite^®^ 2000, Siemens Medical Diagnostic Solutions, Malvern, PA, USA), according to the manufacturer’s instructions.

For the oestradiol-17β (E2) assay, we used a chemical luminescent enzyme in the solid phase (Immulite^®^ 2000 oestradiol immuno-assay). The solid phase was composed of a polyclonal anti-oestradiol antibody (rabbit’s antibody). The reagent contained alkaline phosphatase, conjugated with oestradiol. The conjugated oestradiol-enzymatic competed with the oestradiol in the sample for limited sites bound to antibodies on the solid phase. The sample and the reagent in excess were removed by centrifugal washing. Finally, the substratum’s chemiluminescence was added to the solid phase and a signal in proportion to the binding enzyme was generated. The volume required for every cycle of incubation (1 × 60 min) was 25 μL of serum. It was recommended to re-dose the samples after dilution with superior values of 1200 μL/mL. The precision was valued by measuring the repetition and reproducibility, with a coefficient of variability (CV) of 2.2% intra-assay and 5.1% inter-assay, respectively. The sensitivity of the assay was 9 pg/mL

A sequential competitive immunoassay (Immulite^®^ 2000 progesterone) was used for the progesterone (P4) assay. The volume required for every cycle of incubation (2 × 30 min) was 50 μL of serum or plasma. All samples that had superior levels compared to the calibration range test were diluted before the test, and the corrections related to dilutions were manually calculated. Nevertheless, data obtained using plasma were interpreted with prudence, considering that the EDTA had a meaningful effect on the results. The CV was 5.7% intra-assay and 3.8%, inter-assay, respectively. The sensitivity of the assay was 0.25 ng/mL.

For the cortisol assay, a chemical luminescent enzyme in the solid phase (Immulite^®^ 2000 cortisol immuno-assay) was used. The volume required for every cycle of incubation (2 × 30 min) was 10 μL of serum or plasma. The CV was 0.27% intra-assay and 6.1% inter-assay. The sensitivity of the assay was 0.20 μg/dL.

Analytes (hematochemical and electrolytic parameters) were analyzed by automated spectrophotometry (BT 3500, Biotech Instruments S.p.a., Rome, Italy) using the colorimetric enzymatic method and reagents of the same brand. An indirect potentiometric method was used to analyze Na^+^ and K^+^.

### 2.4. Statistical Analysis

JMP^®^ 16 (SAS Institute Inc., Cary, NC, USA) software was used to perform statistical analyses. For all the parameters, the descriptive statistic was obtained. The influence of lactation phase, breed, and their interaction on the variables were analyzed using a two-way analysis of variance (ANOVA), and multiple comparisons were made by Tukey HSD test. Variables that were not normally distributed were appropriately transformed. The relationships between analytes and hormones were examined by linear regression analysis, and the correlation was expressed by Pearson’s correlation coefficient. Differences were considered to be statistically significant at *p*-value < 0.05.

## 3. Results

The results for each of the measured parameters are presented in Table 1. The data indicate that for some parameters, significant differences exist between different breeds used in this study (Holstein, Brown Swiss, and Modicana) as shown in Table 2. The fact that the lactating Modicana cows, which also have the lowest average daily milk yield, had the lowest blood concentrations of Ca^++^ and Mg^++^, as well as the lowest activity of AST and LDH is highly evident, while the Holsteins had the highest concentrations of Fe++, with the Brown Swiss having the highest ALT activity.

**Figure 1:**
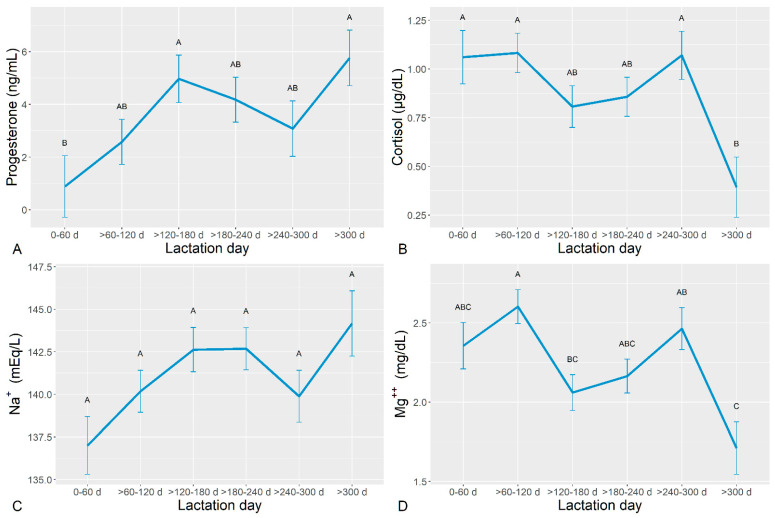
Serum concentrations of progesterone (**A**), cortisol (**B**), Na^+^ (**C**), and Mg^++^ (**D**) along lactation. Different letters indicate significantly different values according to the Tukey–Kramer test.

The circulating blood parameter concentrations are shown in Table 3. The mean ± SE plots of parameters that were significantly affected during the lactation phase, progesterone (A), cortisol (B), Na^+^ (C), and Mg^++^ (D), are presented in Figure 1.

The results indicate that the mean total P4 concentrations undergo a marked increase during the first part of the lactation phase peaking at >120–180 d, followed by a decrease during the next four months, which is followed by a drastic second rise after 300 d (Figure 1A). On the other hand, the mean total cortisol followed an opposite trend, showing a progressive decrease in concentrations, except for the >240 to 300 d, followed by a steep drop at >300 (*p* < 0.0001; Figure 1B).

Mean total Na^+^ concentrations showed the lowest values at 0–60 d and the highest ones at >300 d (*p* = 0.0064; Figure 1C), whereas the mean total Mg^++^ peaked at >60–120 d and was the lowest at >300 d (*p* = 0.0003; Figure 1D).

**Figure 2:**
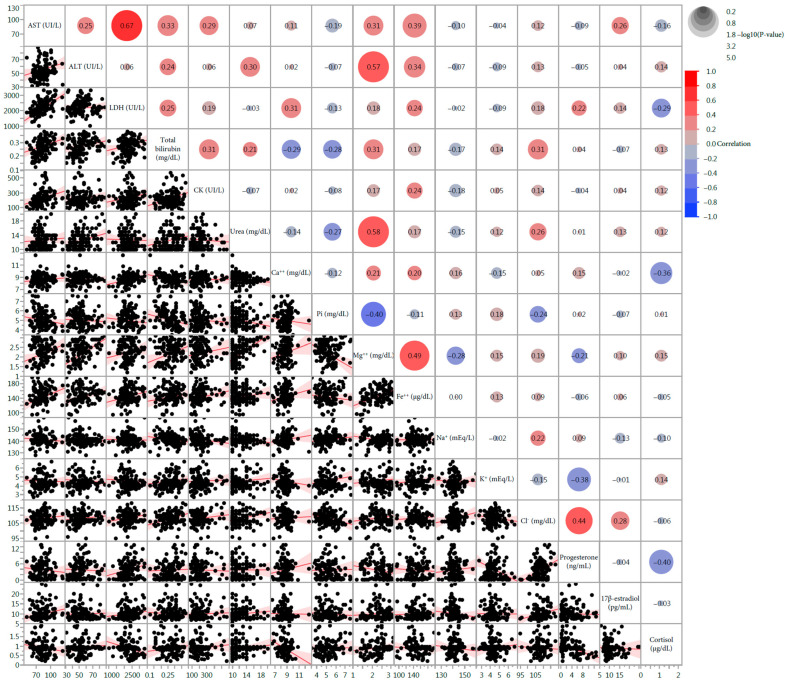
Correlation plot among all the analyzed variables. The size of the circles is proportional to the *p*-values and their color reflects the correlation coefficient (r).

The impact of lactation was reflected by the significant correlations between P4 with cortisol (r = −0.3964, *p* = 0.0010), Cl^−^ (r = 0.4421, *p* = 0.0002), and K^+^, (r = −0.3826, *p* = 0.0013), and the significant correlations between cortisol with Ca^++^ (r = −0.3573, *p* = 0.0035) and LDH (r = −0.2944, *p* = 0.0173). The correlation plot among all the parameters is reported in Figure 2.

## 4. Discussion

The effect of the different lactation phases on the magnitude of fluctuations in the blood serum concentrations of P4, E2, cortisol, electrolytes (Na^+^, Ca^++^, Mg^++^, K^+^, Cl^−^, and P), and some analytes (AST, ALT, LDH, CK, total bilirubin, urea, and Fe^++^) in lactating dairy cows of different breeds have been evaluated as part of a wider research initiative that also delved into the physiological responses in lactating cows.

Along the different lactation phases, all cows registered circulating concentrations of hormonal steroids [5,25,26,27] and analytes [28,29,30] that were in line with known physiological ranges for the bovine species.

### 4.1. Steroid Hormones

The circulating P4 concentrations in the different breeds used in this study did not show significant differences confirming data previously reported by Engida et al. [31]. The highest P4 concentrations were registered during the first part of lactation, followed by a marked decrease during the >240–300 d period, and with another increasing episode after 300 d. This pattern confirms P4’s involvement in the physiological development of the mammary gland and the onset of lactation [4,5]. On the other hand, Sangsritavong et al. [6] reported a rapid decline of P4 occurring immediately after parturition. This apparent discrepancy might be explained by the fact that this study ascribed the sudden and disruptive onset of milk production to the 0–60 d period. Furthermore, it was precisely at this early stage that the lowest concentrations of P4 were detected.

It is possible to presume that P4 is also involved as a regulator of electrolyte homeostasis. This is supported by the positive correlation with Cl- and the negative correlation with K^+^ [32,33]. However, the exact mechanism remains unclear since the absorption of these macroelements through the intestinal wall normally occurs passively without the need for the intervention of any special mechanisms.

The observed flux in blood serum cortisol concentrations is in line with those reported by Svennersten-Sjaunja and Olsson [34]. The significant effect of lactation on cortisol trends confirms the metabolic effect of corticosteroids in their multifunctional roles, also with a direct implication on the mammary gland [35]. Hence, the highest cortisol values observed from 0–60 d to >60–120 d of the lactation period are associated with the mobilization of the cow’s body reserves to meet the high metabolic demands to match the requirements needed during peak lactation. This occurs at 5–7 weeks following calving, since the cow’s ability for maximum feed intake occurs at a later stage, i.e., at 8–20 weeks postpartum leading to a nutritional deficiency that is compensated by the mobilization of body reserves. The primary function of the increases in cortisol is to prevent hypoglycemia during periods of acute and prolonged stress, through mechanisms that influence the energy provision metabolic pathways [35]. This coincides with peak milk production at >60–120 d post-calving, and again during the >240–300 d window when cows are still being milked while the fetus is undergoing an intense growth phase. Therefore, it cannot be ruled out that cortisol’s sudden decline at 300 d is related to the dry period of the cows. Moreover, cortisol is a marker of the degree of stressful events when it is inappropriately high or low. The concentrations observed in this study did not exceed normal ranges indicating a general condition of well-being, as previously recorded also in periparturient [36] and postpartum in dairy cows [37]. No cortisol seasonal fluctuations were detected since the temperature ranges within the barns were kept close to the comfort zone.

The observed negative correlation between P4 and cortisol in lactating dairy cows is in line with previous studies [38,39]. This negative association supports the hypothesis that cortisol is an endogenous inhibitor of the effects of P4; in fact, it is reported that it actually contributes to the conversion of P4 to estrogens [40]. Although no correlations between P4 and E2 were observed, their opposite trends corroborate the hypothesis of the conversion of P4 to estrogens. On the other hand, P4 is considered to be an indirect precursor for the production of cortisol [41], which would be in contradiction with the negative correlation. This may be justified by the plausible existence of a negative feedback mechanism between these two steroid hormones.

The onset of synthesis and secretion of colostrum by dairy cows in the first few days after calving exerts a large demand for Ca^++^ [42] mobilization. In addition, during these first few days postpartum, several homeostatic mechanisms need to adapt to maintain the Ca^++^ plasma pool with respect to its requirements for colostrum and milk [43]. Another significant calcium drain occurs during maximum fetal growth. Hence, the significant negative correlation between cortisol and Ca^++^ could be due to the increased mobility of calcium at the moment of peak milk production and again during maximum fetal growth. This is presumably mirrored by the simultaneous increase in cortisol concentrations during these physiological periods. A key mode of action for the rapid physiological effects of glucocorticoids involves changes in Ca^++^ concentrations [44,45], and studies have shown that this steroid either stimulates or inhibits basal Ca^++^ levels in a tissue-specific manner [46,47]. The adaptation to lactation through Ca-metabolizing pathways is one of the best examples of an efficient interaction of homeostatic and homeorhetic control systems to ensure adequate Ca^++^ supply during instances of acute need. On this basis, it is possible to presume that cortisol seems to be involved in the direct or indirect regulating mechanism for calcium homeostasis during different phases of lactation in dairy cows.

In the same manner, the negative correlation between cortisol concentrations and LDH activity may be affected by several mechanisms including glycogen metabolism. On the one hand, LDH activity is present in all cells where the process of glycolysis takes place [48]; by contrast, glucocorticoids in the liver increase glycogen storage, whereas in skeletal muscle they play a permissive role for catecholamine-induced glycogenolysis and/or inhibit insulin-stimulated glycogen synthesis [49].

### 4.2. Analytes (Na^+^, Cl^−^, K^+^, Fe^++^, Ca^++^, Pi, and Mg^++^)

The metabolic homeostatic control varies dynamically throughout lactation; however, it is important to underline that all the measurements remained consistently within the physiological ranges.

Although differences in average Ca^++^, Mg^++^, and Fe^++^ values across breeds were observed, the lack of significant interactions between breeds and the measured lactation phase parameters suggests that they all have similar behavior during the various lactation phases. Nonetheless, these three parameters registered the lowest values in the Modicana breed, which incidentally also has the lowest genetic potential for average daily milk yield when compared to the other two breeds in this study.

The data indicate that lactation was characterized by an overall increasing trend in the blood serum concentration of Na^+^ that followed the general lactation curve. This observation may be due to the increased demand for Na^+^ to be secreted in milk thereby stimulating the mobilization from body reserves into the blood. Other studies have reported no significant variations during lactation [50] in high-yield dairy cows or during the entire postpartum period [14,27,51]. These observations could be due to the different physiological regulatory modes of electrolyte concentrations in milk that may have relevance to the different adaptive morphological and functional changes in lactating dairy cows. Nevertheless, these changes usually do not exceed fairly broad limits of reference values, as confirmed by Batchelder et al. [52] and Skrzypczak et al. [53] for Na^+^, K^+^, and Cl^−^ in lactating cows.

This study showed a variable trend of Cl^−^ along lactation but with no significant differences among phases. Whereas, Jarosz [54] observed a stable Cl^−^ trend in primiparous lactating cows, the variable trends observed in this study could be explained by the inclusion of multiparous cows. Moreover, although it is well known that the regulation of Cl^−^ concentration in blood is associated with that of Na^+^, no correlation was found between them.

The overall K+ concentration was stable throughout the entire observation period, without any deviation from the physiological range. Similar constant concentrations were previously reported for the first months of lactation by Grünwaldt et al. [55], Nozad et al. [56], and Sattler et al. [57].

The observed fluctuations in Fe^++^ at the different lactation phases suggest a potential “oestrogen-iron” axis that involves iron metabolism in response to hematologic (erythropoiesis) and non-hematologic (lactation) needs for iron [7,11] in lactating dairy cows. The significantly higher Fe^++^ concentration in Holstein cows calls for a deeper study that delves into iron metabolism in breeds other than the Holstein.

The initiation of lactogenesis following parturition is the driving force behind the changes in Mg^++^, Pi, and Ca^++^ levels [58]. In this study, Mg^++^ concentrations were significantly lower at the end of lactation when compared to the early or middle lactation phases. This demonstrates different cow requirements for Mg^++^ in line with its particular physiological phases. In fact, lactating cows require magnesium at 0.20% of the diet dry matter and 0.12% for the gestating phase [59]. The significant decrease observed at >300 d could be an expression of a prior adaptive response at the end of lactation, predisposing the cow to future rapid and exponential Mg^++^ decline in postpartum within 2 to 3 d to the normal values, as recorded by Shappell et al. [60]. The highest Mg^++^ concentrations were observed in both Brown and Holstein, which could be related to the higher demand for this element in cows genetically predisposed to high milk yields.

With regard to the Ca^++^, a trend of a progressively increasing concentration was observed along the lactation period, with significantly higher mean values in the Brown Swiss and Holstein cows. As described for Mg^++^, this is probably due to the increased ability to mobilize calcium from bones in high milk-yielding dairy cows, which is presumably mirrored by the constant increase in serum calcium concentrations along the lactation phases. The use of anionic salts in combination with adequate Ca^++^ and Mg^++^ supplementation may help improve feed dry matter intakes and reduce the negative energy balance in the post-calving period while preventing hypocalcemia. Nevertheless, it should be noted that clinical hypomagnesemia and hypocalcemia were absent in the current study, as Mg^++^ and Ca^++^ concentrations remained consistently within the physiological range [61].

### 4.3. Hematochemical Analytes (AST, ALT, LDH, CK, Total Bilirubin, Urea)

Changes in the intensity of metabolic processes during the lactation cycle in dairy cows are reflected as variations in the concentration of hematochemical blood analytes. However, although this study reported differences in average AST, ALT, and LDH activities across the three breeds, all cows responded similarly to lactation phases. This confirms previous data recorded in bulls with regard to genotype [62], calves [63], beef cattle [55,64], and lactating dairy cows [65,66].

AST is usually used as an indicator of liver or muscle injury; hence, deviations from normal state values can be attributed to its increased activity in cells, especially liver cells, but can also be an indication of cell structure damage in clinically healthy dairy cows during lactation and in the dry period [67]. ALT activity in cattle, goats, and sheep is not specifically linked to the liver [68] and can be influenced by age and muscle exercise [69]. Ruminant liver cells do not usually show high ALT activity, and the increased activity of that enzyme in the serum during liver damage, even in necrosis, is insignificant [70].

In the case of hepatic or muscle enzymes, the values for AST and ALT activity were in line with the normal ranges found by Doornenbal et al., Gonano et al., and Bionda et al. [21,71,72] for beef heifers and by Moretti et al. [30] for cows of various breeds. However, the results indicated a significantly higher level in Brown and Holstein breeds, most probably linked to their higher milk yields. On this basis, although AST activity is an indicator of liver or muscle injury, it is also possible to consider that changes can be the consequence of its increased physiological activity in cells, especially hepatic cells, but also a reflection of cell structure damage in clinically healthy dairy cows during lactation and in the dry period, as previously recorded by Stojević et [67]. According to Tainturier et al. [71], the activity of the AST enzyme may occasionally show small irregular changes during early lactation, which, in our case, extend into subsequent lactation phases.

The non-significant changes in ALT activity along the different lactation phases are not in agreement with previous findings reporting that the lowest activity occurs during early lactation [71]. In addition, ALT activity in cattle, goats, and sheep is not specific to the liver [72] and is also influenced by age and muscle exercise [73]. The high activities observed in the Brown and Holstein breeds do not correlate with the aforementioned variables, since the cows in this study were homogenous for age and subjected to the same management practices.

The observed LDH trend is in partial agreement with data recorded by Asmare et al. [74] in dairy cows at different stages of milk production. In addition, Asmare et al. [74] reported that the activity of the LDH1 isoenzyme was found to be significantly higher in late pregnant cows than in early lactating cows. Although LDH isoenzyme activities were not assayed in this investigation, the trend of total LDH showed that its activity was highest in middle-lactating cows followed by cows at the late-lactating stage.

CK is present and active in all smooth muscles [75] and its isoenzymes are coupled with many cellular energetic processes. The CK activity in this study was higher than those observed in most studies but still within the range set by Latimer [76] for cows and beef heifers. The data from this study indicate that the highest CK activity occurred at the onset of lactation and again at >240–300 d, with a slump at >180–240 and >300 d. This could be due to muscle restoration, confirming that CK represents an indicator of muscle protein turnover associated with energy utilization [77]. Despite the energetic flux being much lower in smooth muscle when compared to skeletal muscle, the data obtained confirmed that CK is present and active in all smooth muscles [75]. On this basis, it is possible to justify its significant increase in conjunction with mammary gland development and lactation initiation, and likewise during uterine volume growth and increased fetal activity. Conversely, it is possible that its decreases during these phases can be attributed to limited fetal activity and development and the onset of the dry phase. This corroborates the high degree of plasticity of the smooth mammary muscle that undergoes transformation during the different stages of lactation. In addition, although CK activity can vary according to the time of the day, age, growth rate, and physiological status [78], the present study excluded the effect of these variables because the animals were homogenous for age and physiological conditions, and the blood samples were always collected at the same time of the day.

The total bilirubin concentration remained within the physiological range along the different lactating phases, showing a balanced metabolism that contrasts with the notion that an increase in concentrations coincides with a negative energy balance that can affect both fat mobilization and liver function [79,80,81].

The recorded values of urea were in line with the range found by Doornenbal et al. [71] and Gonano et al. [72] in beef heifers, by Moretti et al. [30] in cows, and by Andjelić et al. [82] in lactating dairy cows, who also reported a decrease in urea, which aligns with the observations made in the present study at >300 d of lactation. This non-significant reduction in the urea concentration could reflect the lower protein ingestion occurring at the end of lactation due to the reduced dry matter intake, as previously described after calving in dairy cows [83], or to the increased recycling of ruminal urea for protein synthesis at the start of lactation as suggested by Quiroz-Rocha et al. [84].

Finally, it is possible to presume that during lactation, even if the homeostatic processes persist, healthy cows mobilize their tissue reserves for milk production and may lose appreciable amounts of body weight and muscle. It seems likely that the amplitude of urea changes in lactating dairy cows are functional and reversible and related to the metabolic demands of specific phases of lactation in the assessment of metabolic health in dairy herds.

## 5. Conclusions

The most significant outcomes of this study are the following:

(1) Lactation induces significant changes in the blood serum concentrations of P4, cortisol, Na^+^, and Mg^++^;
(2) The breeds used in this study showed a significant effect on AST, ALT, and LDH activities and the concentration of Ca^++^, Mg^++^; and Fe^++^, most probably related to the milking yield potential of the breed, but the variations throughout the lactation period were similar for all the breeds;
(3) Significant correlations were recorded for P4 with cortisol, Cl^−^,and K^+^ and for cortisol with Ca^++^ and LDH.

The observed results in this study confirmed that at the onset of lactation and throughout the lactation period, there is a dynamic crosstalk between steroid hormones and hematochemical analytes. Hence, the priority of the lactating animal is to provide the mammary gland with nutrients via metabolic changes orchestrated by several mechanisms, among which the endocrine system, and more specifically, the steroid hormones can be considered as being the main driving force. The only critical point of the study is the small group size, which was due to the characteristics of the farm involved. However, this limitation was offset by the homogeneity of the different breed groups, represented by both homogeneous age, BCS, and stage of lactation, and the same feed given throughout the entire trial period, in order to minimize the interference of several internal and external factors.

Since it seems clear that any breed effect will be linked to milk yield, the physiological differences among breeds should be considered for a correct interpretation of the laboratory diagnostics in the framework of endocrine and analytes monitoring of dairy cows, according to early, middle, and late lactation phases. Understanding the differences among breeds might improve their management nutrition and productivity in commercial dairy farms. Further research would be desirable to broaden and deepen the adaptive strategies adopted by different dairy breeds to cope with the physiological changes occurring at different lactation phases.

## Figures and Tables

**Table 1:** *p*-values indicating the significance of the effect of Lactation phase and Breed factors and their interaction (Lactation Phase × Breed) on the analyzed blood parameters in lactating cows of Holstein, Brown Swiss, and Modicana breeds and R^2^ of the models. *p*-values are reported in bold when significant (*p* < 0.05).

	Lactation Phase	Breed	Lactation Phase × Breed	R^2^
**Progesterone (ng/mL)**	**0.0166**	0.2022	0.9877	0.29
**17β-estradiol (pg/mL)**	0.1141	0.9186	0.6323	0.27
**Cortisol (μg/dL)**	**0.0074**	0.1110	0.9416	0.37
**Ca^++^ (mg/dL)**	0.4525	**0.0064**	0.9574	0.27
**Pi (mg/dL)**	0.6123	0.0901	0.4070	0.30
**Mg^++^ (mg/dL)**	**0.0003**	**0.0024**	0.5275	0.56
**Fe^++^ (μg/dL)**	0.2396	**0.0002**	0.4080	0.44
**Na^+^ (mEq/L)**	0.0414	0.1317	0.2354	0.35
**K^+^ (mEq/L)**	0.2653	**0.0457**	0.1647	0.34
**Cl^−^ (mg/dL)**	0.1766	0.2066	0.2128	0.34
**AST (UI/L)**	0.5437	**0.0042**	0.5502	0.33
**ALT (UI/L)**	0.3022	**0.0084**	0.4277	0.24
**LDH (UI/L)**	0.0659	**<0.0001**	0.1478	0.56
**Total bilirubin (mg/dL)**	0.1044	0.3581	0.5391	0.26
**CK (UI/L)**	0.5270	0.3071	0.5625	0.29
**Urea (mg/dL)**	0.1135	0.5938	0.2517	0.31

**Table 2:** Mean and standard error (SE) of the analyzed blood parameters in lactating cows of different breeds. The parameters are reported in bold when a significant effect of the “Breed” factor was found.

		Holstein	Brown Swiss	Modicana
**Average daily milk yield** (Mean ± SD)		28.8 ± 3.3 kg	27.3 ± 3.1 kg	15.0 ± 2.0 kg
**Progesterone (ng/mL)**	Mean ± SE	3.5 ± 0.48	4.12 ± 0.64	4.04 ± 0.68
**17β-oestradiol (pg/mL)**	Mean ± SE	10.00 ± 0.64	10.29 ± 0.91	9.82 ± 0.51
**Cortisol (μg/dL)**	Mean ± SE	0.79 ± 0.07	0.84 ± 0.10	1.01 ± 0.08
**Ca^++^ (mg/dL)**	Mean ± SE	**8.91 ± 0.13 ^A^**	**8.96 ± 0.12 ^A^**	**8.25 ± 0.19 ^B^**
**Pi (mg/dL)**	Mean ± SE	4.93 ± 0.13	4.81 ± 0.12	5.24 ± 0.14
**Mg^++^ (mg/dL)**	Mean ± SE	**2.31 ± 0.10 ^A^**	**2.43 ± 0.11 ^A^**	**1.98 ± 0.08 ^B^**
**Fe^++^ (μg/dL)**	Mean ± SE	**157.50 ± 4.08 ^A^**	**142.28 ± 3.87 ^B^**	**135.67 ± 3.48 ^B^**
**Na^+^ (mEq/L)**	Mean ± SE	140.05 ± 1.19	142.39 ± 1.18	141.03 ± 0.92
**K^+^ (mEq/L)**	Mean ± SE	**4.77 ± 0.19 ^A^**	**4.40 ± 0.13 ^A^**	**4.44 ± 0.12 ^A^**
**Cl^−^ (mg/dL)**	Mean ± SE	107.09 ± 0.85	109.00 ± 0.70	108.87 ± 0.92
**AST (UI/L)**	Mean ± SE	**90.95 ± 2.43 ^A^**	**90.72 ± 3.74 ^A^**	**80.37 ± 2.44 ^B^**
**ALT (UI/L)**	Mean ± SE	**53.00 ± 2.28 ^A^**	**62.94 ± 3.16 ^A^**	**53.03 ± 2.35 ^B^**
**LDH (UI/L)**	Mean ± SE	**2314.24 ± 87.16 ^A^**	**2467.61 ± 92.46 ^A^**	**1945.53 ± 65.86 ^B^**
**Total bilirubin (mg/dL)**	Mean ± SE	0.28 ± 0.01	0.27 ± 0.01	0.28 ± 0.01
**CK (UI/L)**	Mean ± SE	244.33 ± 20.91	199.89 ± 15.91	233.97 ± 22.88
**Urea (mg/dL)**	Mean ± SE	12.95 ± 0.52	13.17 ± 0.66	12.10 ± 0.34

Within each row, different superscript letters indicate that that parameter shows statistically significant differences between breeds based on the Tukey–Kramer test *(p* < 0.05).

**Table 3:** Mean and standard error (SE) of the analyzed blood parameters in different phases of lactation. The parameters are reported in bold when a significant effect of the “Lactation phase” factor was found (see Table 1).

Lactation Phase (Days)	0–60	>60–120	>120–180	>180–240	>240–300	>300
**Progesterone (ng/mL)**	1.01 ± 0.43 ^B^	2.98 ± 0.88 ^AB^	5.35 ± 1.04 ^A^	3.74 ± 0.71 ^AB^	2.95 ± 0.76 ^AB^	5.28 ± 0.43 ^A^
**17β-oestradiol (pg/mL)**	10.45 ± 2.35	10.12 ± 0.53	9.31 ± 0.42	8.67 ± 0.36	11.96 ± 1.38	11.46 ± 1.39
**Cortisol (μg/dL)**	1.04 ± 0.10 ^A^	1.09 ± 0.08 ^A^	0.84 ± 0.10 ^AB^	0.89 ± 0.06 ^AB^	1.00 ± 0.20 ^A^	0.42 ± 0.04 ^B^
**Ca^++^ (mg/dL)**	8.03 ± 0.27	8.65 ± 0.33	8.46 ± 0.16	8.68 ± 0.19	8.74 ± 0.18	8.94 ± 0.32
**Pi (mg/dL)**	4.96 ± 0.44	4.87 ± 0.17	5.03 ± 0.16	5.23 ± 0.17	4.73 ± 0.16	5.23 ± 0.22
**Mg^++^ (mg/dL)**	2.40 ± 0.20 ^ABC^	2.55 ± 0.08 ^A^	2.01 ± 0.08 ^BC^	2.00 ± 0.16 ^ABC^	2.51 ± 0.08 ^AB^	1.86 ± 0.15 ^C^
**Fe^++^ (μg/dL)**	143.29 ± 4.51	145.80 ± 3.31	142 ± 5.66	144.20 ± 6.29	153.33 ± 7.80	132.13 ± 9.24
**Na+ (mEq/L)**	137.43 ± 2.10 ^A^	4.77 ± 0.19 ^A^	142.36 ± 0.90 ^A^	143.47 ± 1.37 ^A^	140 ± 1.18 ^A^	141.63 ± 2.24 ^A^
**K^+^ (mEq/L)**	4.77 ± 0.26	4.77 ± 0.19	4.46 ± 0.09	4.47 ± 0.20	4.80 ± 0.31	4.19 ± 0.08
**Cl^−^ (mg/dL)**	105.86 ± 2.76	107.09 ± 0.85	109.86 ± 1.51	106.73 ± 0.67	109.78 ± 1.19	108.50 ± 0.94
**AST (UI/L)**	82.86 ± 4.89	90.95 ± 2.43	90.36 ± 4.09	83.13 ± 3.67	89.11 ± 4.51	86.63 ± 6.05
**ALT (UI/L)**	61.14 ± 7.06	53.00 ± 2.28	53.57 ± 2.00	56.00 ± 3.84	56.89 ± 3.44	46.50 ± 2.69
**LDH (UI/L)**	1930.57 ± 117.48	2314.24 ± 87.16	2353.14 ± 96.20	2095.47 ± 99.76	2364.00 ± 160.62	2354.43 ± 253.91
**Total bilirubin (mg/dL)**	0.26 ± 0.02	0.28 ± 0.01	0.29 ± 0.02	0.28 ± 0.01	0.29 ± 0.02	0.23 ± 0.02
**CK (UI/L)**	217.71 ± 21.34	244.33 ± 20.91	294.57 ± 43.06	186.43 ± 20.20	243.78 ± 18.81	185.38 ± 18.47
**Urea (mg/dL)**	13.00 ± 0.87	12.95 ± 0.52	11.71 ± 0.29	12.67 ± 0.85	13.44 ± 0.65	12.25 ± 0.53

Within each row, different superscript letters indicate that that parameter shows statistically significant differences between lactation phases based on the Tukey–Kramer test (*p* < 0.05).

## Data Availability

The original contributions presented in the study are included in the article; further inquiries can be directed to the corresponding author.

## Supplementary Materials

The following supporting information can be downloaded at: https://www.mdpi.com/article/10.3390/ani14223336/s1, Table S1: Ingredients and chemical composition of concentrate and hay used during the trial; Table S2: Mean ± SD temperature and humidity recorded daily in Ragusa (https://www.wunderground.com/dashboard/pws/IRAGUSAD2), where the cows included in the present study were bred, and related Temperature Humidity Index (THI). To limit heat stress, during the hot period pasture was not available and the livestock housing was equipped with automatic system fans and freely accessible showers.

## References

[1] Spaans O.K., Kuhn-Sherlock B., Hickey A., Crookenden M.A., Heiser A., Burke C.R., Phyn C.V.C., Roche J.R. (2022). Temporal profiles describing markers of inflammation and metabolism during the transition period of pasture-based, seasonal-calving dairy cows. J. Dairy Sci..

[2] Walter L.L., Gärtner T., Gernand E., Wehrend A., Donat K. (2022). Effects of parity and stage of lactation on trend and variability of metabolic markers in dairy cows. Animals.

[3] Bruckmaier R.M., Gross J.J. (2017). Lactational challenges in transition dairy cows. Anim. Prod. Sci..

[4] Kindahl H., Kornmatitsuk B., Gustafsson H. (2004). The cow in endocrine focus before and after calving. Reprod. Domest. Anim..

[5] Kindahl H., Kornmatitsuk B., Königsson K., Gustafsson H. (2002). Endocrine changes in late bovine pregnancy with special emphasis on fetal well-being. Domest. Anim. Endocrinol..

[6] Sangsritavong S., Combs D.K., Sartori R., Armentano L.E., Wiltbank M.C. (2002). High feed intake increases liver blood flow and metabolism of progesterone and estradiol-17beta in dairy cattle. J. Dairy Sci..

[7] Yang X., Xu M., Wang J., Xie J. (2016). Effect of estrogen on iron metabolism in mammals. Sheng Li Xue Bao [Acta Physiol. Sin.].

[8] Satué K., Fazio E., Cravana C., Medica P. (2023). Hepcidin, ferritin and iron homeostasis in pregnant Spanish Purebred mares. Theriogenology.

[9] Satué K., Fazio E., La Fauci D., Medica P. (2023). Changes of Hepcidin, Ferritin and Iron levels in cycling Purebred Spanish Mares. Animals.

[10] Satué K., Fazio E., Medica P. (2023). Estrogen-iron axis in cyclic mares: Effect of age. Theriogenology.

[11] Hamad M., Bajbouj K., Taneera J. (2020). The Case for an Estrogen-iron Axis in Health and Disease. Exp. Clin. Endocrinol. Diabetes.

[12] Qian Y., Zhang S., Guo W., Ma J., Chen Y., Wang L., Zhao M., Liu S. (2015). Polychlorinated biphenyls (PCBs) inhibit hepcidin expression through an estrogen-like effect associated with disordered systemic iron homeostasis. Chem. Res. Toxicol..

[13] Dobson H., Ghuman S., Prabhakar S., Smith R. (2003). A conceptual model of the influence of stress on female reproduction. Reproduction.

[14] Kupczyński R., Chudoba-Drozdowska B. (2002). Values of selected biochemical parameters of cows’ blood during their drying-off and the beginning of lactation. Electron. J. Pol. Agric. Univ..

[15] Liggins G.C. (1994). The role of cortisol in preparing the fetus for birth. Reprod. Fertil. Dev..

[16] Nathanielsz P.W. (1993). A time to be born: How the fetus signals to the mother that it is time to leave the uterus. Cornell Vet..

[17] Dang A.K., Jamwal M., Kaur M., Kimothi S.P., Pal S., De K., Pathan M.M., Swain D.K., Mohapatra S.K., Kapila S. (2013). Effect of micronutrient supplementation around calving on the plasma cortisol levels of Murrah buffaloes and Sahiwal and Karan Fries cows. Trop. Anim. Health Prod..

[18] Fiore E., Barberio A., Morgante M., Rizzo M., Giudice E., Piccione G., Lora M., Gianesella M. (2015). Glucose infusion response to some biochemical parameters in dairy cows during the transition period. Anim. Sci. Pap. Rep..

[19] Grala T.M., Kuhn-Sherlock B., Roche J.R., Jordan O.M., Phyn C.V.C., Burke C.R., Meier S. (2022). Changes in plasma electrolytes, minerals, and hepatic markers of health across the transition period in dairy cows divergent in genetic merit for fertility traits and postpartum anovulatory intervals. J. Dairy Sci..

[20] Bellová V., Pechová A., Dvořák R., Pavlata L. (2009). Influence of full-fat soybean seeds and hydrolyzed palm oil on the metabolism of lactating dairy cows. Acta Vet. Brno.

[21] Bionda A., Lopreiato V., Crepaldi P., Chiofalo V., Fazio E., Oteri M., Amato A., Liotta L. (2022). Diet supplemented with olive cake as a model of circular economy: Metabolic response in beef cattle. Front. Sustain. Food Syst..

[22] Fazio E., Bionda A., Chiofalo V., La Fauci D., Randazzo C., Pino A., Crepaldi P., Attard G., Liotta L., Lopreiato V. (2023). Effects of dietary enrichment with olive cake on the thyroid and adrenocortical responses in growing beef calves. Animals.

[23] Bertoni G., Trevisi E., Han X., Bionaz M. (2008). Effects of inflammatory conditions on liver activity in puerperium period and consequences for performance in dairy cows. J. Dairy Sci..

[24] National Research Council (U.S.) (1971). Committee on Physiological Effects of Environmental Factors on Animals. Guide to Environmental Research on Animals.

[25] Astiti L.G.S., Panjaitan T. (2013). Serum Progesterone concentration in Bali cow during pregnancy. Open Sci. Repos. Vet. Med. Online.

[26] Duong H.T., Piotrowska-Tomala K.K., Acosta T.J., Bah M.M., Sinderewicz E., Majewska M., Jankowska K., Okuda K., Skarzynski D.J. (2012). Effects of cortisol on pregnancy rate and corpus luteum function in heifers: An in vivo study. J. Reprod. Dev..

[27] Yanhong Y., Yuin C., Sijiu Y. Histological characteristics of corpus luteum in Yak during early pregnancy. Proceedings of the Fourth International Congress on Yak.

[28] Dodamani M.S., Mohteshamuddin K., Awati S.D., Tandle M.K., Honnappagol S.S. (2009). Evaluation of serum profile during various stages of gestation in crossbred Deoni Cows. Vet. World.

[29] Knight C.H. (2001). Lactation and gestation in dairy cows: Flexibility avoids nutritional extremes. Proc. Nutr. Soc..

[30] Moretti P., Paltrinieri S., Trevisi E., Probo M., Ferrari A., Minuti A., Giordano A. (2017). Reference intervals for hematological and biochemical parameters, acute phase proteins and markers of oxidation in Holstein dairy cows around 3 and 30 days after calving. Res. Vet. Sci..

[31] Engida T., Lobago F., Lemma A., Yenehun A.M., Mekete B. (2022). Validation of the Human Progesterone Assay Kit for Cattle as a Pregnancy Diagnosis Tool. Vet. Med. Int..

[32] Myles K., Funder J. (1996). Progesterone binding to mineralocorticoid receptors: In vitro and in vivo studies. Am. J. Physiol. Endocrinol. Metab..

[33] Wenner M., Stachenfeld N., Hackey A.C. (2017). Sex hormones and environmental factors affecting exercise. Sex Hormones, Exercise and Women.

[34] Svennersten-Sjaunja K., Olsson K. (2005). Endocrinology of milk production. Domest. Anim. Endocrinol..

[35] Benfield R.D., Newton E.R., Tanner C.J., Heitkemper M.M. (2014). Cortisol as a biomarker of stress in term human labor: Physiological and methodological issues. Biol. Res. Nurs..

[36] Guo J., Peters R.R., Kohn R.A. (2007). Effect of a transition diet on production performance and metabolism in periparturient dairy cows. J. Dairy Sci..

[37] Gladden N., McKeegan D., Viora L., Ellis K.A. (2018). Postpartum ketoprofen treatment does not alter stress biomarkers in cows and calves experiencing assisted and unassisted parturition: A randomised controlled trial. Vet. Rec..

[38] Echternkamp S.E. (1984). Relationship between LH and cortisol in acutely stressed beef cows. Theriogenology.

[39] Tallo-Parra O., Carbajal A., Monclús L., Manteca X., Lopez-Bejar M. (2018). Hair cortisol and progesterone detection in dairy cattle: Interrelation with physiological status and milk production. Domest. Anim. Endocrinol..

[40] Kurpińska A., Skrzypczak W. (2020). Hormonal changes in dairy cows during periparturient period. Acta Sci. Pol. Zootech..

[41] Baulieu E.E., Robel P., Schumacher M. (2001). Neurosteroids: Beginning of the story. Int. Rev. Neurobiol..

[42] Martinez N., Risco C.A., Lima F.S., Bisinotto R.S., Greco L.F., Ribeiro E.S., Maunsell F., Galvão K., Santos J.E.P. (2012). Evaluation of peripartal calcium status, energetic profile, and neutrophil function in dairy cows at low or high risk of developing uterine disease. J. Dairy Sci..

[43] Goff J.P. (2008). The monitoring, prevention, and treatment of milk fever and subclinical hypocalcemia in dairy cows. Vet. J..

[44] Chen S., Wang X.H., Zhang X.Z., Wang W.C., Liu D.W., Long Z.Y., Dai W., Chen Q., Xu M.H., Zhou J.H. (2011). High-dose glucocorticoids induce decreases calcium in hypothalamus neurons via plasma membrane Ca(2+) pumps. Neuroreport.

[45] Kim M.H., Lee G.S., Jung E.M., Choi K.C., Oh G.T., Jeung E.B. (2009). Dexamethasone differentially regulates renal and duodenal calcium-processing genes in calbindin-D9k and -D28k knockout mice. Exp. Physiol..

[46] Joëls M., Karst H. (2012). Corticosteroid effects on calcium signaling in limbic neurons. Cell Calcium.

[47] Panettieri R.A., Schaafsma D., Amrani Y., Koziol-White C., Ostrom R., Tliba O. (2019). Non-genomic Effects of Glucocorticoids: An Updated View. Trends Pharmacol. Sci..

[48] Sobiech P., Kuleta Z., Jalynski M. (2002). Serum LDH isoenzyme activity in dairy and beef cows. Acta Sci. Pol. Med. Vet..

[49] Kuo T., McQueen A., Chen T.C., Wang J.C. (2015). Regulation of Glucose Homeostasis by Glucocorticoids. Adv. Exp. Med. Biol..

[50] Safwate A., Davicco M.J., Barlet J.P., Delost P. (1981). Sodium and potassium in blood and milk and plasma aldosterone levels in high-yield dairy cows. Reprod. Nutr. Dev..

[51] Yokus B., Cakir U.D. (2006). Seasonal and physiological variations in serum chemistry and mineral concentrations in cattle. Biol. Trace Elem. Res..

[52] Batchelder C.A., Bertolini M., Mason J.B., Moyer A.L., Hoffert K.A., Petkov S.G., Famula T.R., Angelos J., George L.W., Anderson G.B. (2007). Perinatal physiology in cloned and normal calves: Hematologic and biochemical profiles. Cloning Stem Cells.

[53] Skrzypczak W., Kurpinska A., Stanski L., Jarosz A. (2014). Sodium, potassium and chloride homeostasis in cows during pregnancy and first months of lactation. Acta Biol. Cracoviensia Ser. Zool..

[54] Jarosz A. (2013). Identification of Proteins with Variable Expression in Plasma Proteome of Heifers Before Insemination and During Pregnancy.

[55] Grünwaldt E.G., Guevara J.C., Estévez O.R., Vicente A., Rousselle H., Alcuten N., Aguerregaray D., Stasi C.R. (2005). Biochemical and haematological measurements in beef cattle in Mendoza plain rangelands (Argentina). Trop. Anim. Health Prod..

[56] Nozad S., Ramin A.-G., Moghadam G., Asri-Rezaei S., Babapour A., Ramin S. (2012). Relationship between blood urea, protein, creatinine, triglycerides and macro-mineral concentrations with the quality and quantity of milk in dairy Holstein cows. Vet. Res. Forum.

[57] Sattler N., Fecteau G., Couture Y., Tremblay A. (2001). [Determination of the potassium balances in dairy cows and the examination of daily and lactation period-associated variations]. Can. Vet. J..

[58] DeGaris P.J., Lean I.J. (2008). Milk fever in dairy cows: A review of pathophysiology and control principles. Vet. J..

[59] Martens H., Stumpff F. (2019). Assessment of magnesium intake according to requirement in dairy cows. J. Anim. Physiol. Anim. Nutr..

[60] Shappell N.W., Herbein J.H., Deftos L.J., Aiello R.J. (1987). Effects of dietary calcium and age on parathyroid hormone, calcitonin and serum and milk minerals in the periparturient dairy cow. J. Nutr..

[61] Martens H., Leonhard-Marek S., Röntgen M., Stumpff F. (2018). Magnesium homeostasis in cattle: Absorption and excretion. Nutr. Res. Rev..

[62] Głowiñska B., Oler A. (2013). Biochemical and hormonal characteristics of peripheral blood in bulls in relation to genotype. Folia Biol..

[63] Singh A., Srinivas B. (2020). Plasticity of gut and metabolic limitations of Deoni calves in comparison to crossbred calves on a high plane of nutrition. Trop. Anim. Health Prod..

[64] Boonprong S., Sribhen C., Choothesa A., Parvizi N., Vajrabukka C. (2007). Blood biochemical profiles of Thai indigenous and simmental × brahman crossbred cattle in the central Thailand. J. Vet. Med. A Physiol. Pathol. Clin. Med..

[65] Antanaitis R., Malašauskienė D., Televičius M., Juozaitiene V., Rutkauskas A., Palubinskas G. (2020). Inline changes in lactate dehydrogenase, milk concentration according to the stage and number of lactation periods, including the status of reproduction and milk yield in dairy cows. Pol. J. Vet. Sci..

[66] Marai I.F.M., Habeeb A.A.M., Farghaly H.M. (1999). Productive, physiological and biochemical changes in imported and locally born Friesian and Holstein lactating cows under hot summer conditions of Egypt. Trop. Anim. Health Prod..

[67] Stojević Z., Piršljin J., Milinković-Tur S., Zdelar-Tuk M., Ljubić B.B. (2005). Activities of AST, ALT and GGT in clinically healthy dairy cows during lactation and in the dry period. Vet. Arhiv..

[68] Kramer J.W., Hoffman W.E., Kaneko J.J., Harvey J.W., Bruss M.L. (1997). Clinical Enzymology. Clinical Biochemistry of Domestic Animals.

[69] Scheck K., Weigert P., Lemmer B., Noreisch W. (1980). [Laboratory diagnostic studies of Haflinger horses and mules (pack-animals of the West German Army). 3. Substrates in serum]. Tierarztl. Prax..

[70] Forenbacher S. (1983). Klinička Patologija Probave i Mijene Tvari Domaćih Životinja.

[71] Doornenbal H., Tong A.K., Murray N.L. (1988). Reference values of blood parameters in beef cattle of different ages and stages of lactation. Can. J. Vet. Res..

[72] Gonano C.V., Montanholi Y.R., Schenkel F.S., Smith B.A., Cant J.P., Miller S.P. (2014). The relationship between feed efficiency and the circadian profile of blood plasma analytes measured in beef heifers at different physiological stages. Animal.

[73] Tainturier D., Braun J.P., Rico A.G., Thouvenot J.P. (1984). Variations in blood composition in dairy cows during pregnancy and after calving. Res. Vet. Sci..

[74] Asmare A., Kovac G., Reichel P., Buleca J., Scurokova E. (1998). Serum isoenzyme activity of lactate dehydrogenase in dairy cows at different stages of milk production. Folia Vet..

[75] Clark J.F. (1994). The creatine kinase system in smooth muscle. Mol. Cell. Biochem..

[76] Latimer K.S. (2011). Duncan and Prasse’s Veterinary Laboratory Medicine: Clinical Pathology.

[77] Flück M., Hoppeler H. (2003). Molecular basis of skeletal muscle plasticity--from gene to form and function. Rev. Physiol. Biochem. Pharmacol..

[78] Okonkwo J.C.C., Omeje I.S., Okonkwo I.F.F., Umeghalu I.C.E.C.E. (2010). Effects of breed, sex and source within breed on the blood bilirubin, cholesterol and glucose concentrations of Nigerian goats. Pak. J. Nutr..

[79] Hisadomi S., Haruno A., Fujieda T., Sugino T., Oba M. (2022). Effects of rumen-protected glutamate supplementation during the periparturient period on digestibility, inflammation, metabolic responses, and performance in dairy cows. J. Dairy Sci..

[80] Marcato F., van den Brand H., Jansen C.A., Rutten V.P.M.G., Kemp B., Engel B., Wolthuis-Fillerup M., van Reenen K. (2021). Effects of pre-transport diet, transport duration and transport condition on immune cell subsets, haptoglobin, cortisol and bilirubin in young veal calves. PLoS ONE.

[81] Mayasari N., Trevisi E., Ferrari A., Kemp B., Parmentier H.K., van Knegsel A.T.M. (2019). Relationship between inflammatory biomarkers and oxidative stress with uterine health in dairy cows with different dry period lengths. Transl. Anim. Sci..

[82] Andjelić B., Djoković R., Cincović M., Bogosavljević-Bošković S., Petrović M., Mladenović J., Čukić A. (2022). Relationships between milk and blood biochemical parameters and metabolic status in dairy cows during lactation. Metabolites.

[83] Bertoni G., Trevisi E. (2013). Use of the liver activity index and other metabolic variables in the assessment of metabolic health in dairy herds. Vet. Clin. N. Am. Food Anim. Pract..

[84] Quiroz-Rocha G.F., LeBlanc S.J., Duffield T.F., Wood D., Leslie K.E., Jacobs R.M. (2009). Reference limits for biochemical and hematological analytes of dairy cows one week before and one week after parturition. Can. Vet. J..

