# Supplementary material for: Effect of the Lactation Phases on the Amplitude of Variation in Blood Serum Steroid Hormones and Some Hematochemical Analytes in Three Dairy Cow Breeds
Source: Animals (Basel). 2024 Nov 20;14(22):3336. doi: 10.3390/ani14223336 (PMC11591377; doi:10.3390/ani14223336)
Supplement: Supplementary file 1 [file animals-14-03336-s001.zip › Table S1.pdf]

**Table S1**. Ingredients and chemical composition of concentrate and hay used during the trial.

| **Ingredients, % of DM** | **Concentrate** | **Hay** |
|---|---|---|
| Corn meal | 38 | |
| Roasted soybean flour | 20 | |
| Barley meal | 10 | |
| Beetpulp | 3 | |
| Enriched Olive Cake | 8 | |
| Wheat bran | 6 | |
| Sunflower meal | 5.5 | |
| Rumen Bypass Fat | 1.5 | |
| Minerals and Vitamins Mix | 1.5 | |
| Calcium Carbonate | 1.3 | |
| Saccharomyces dried yeast | 1 | |
| Cane molasses | 1 | |
| Na bicarbonate | 1 | |
| Na chloride | 0.7 | |
| P dicalcium | 0.6 | |
| NutriGen 40 C | 0.5 | |
| Mg oxide | 0.4 | |
| **Chemical composition, % of DM** | | |
| Crude protein | 19.70 | 6.77 |
| Fat | 5.50 | 1.37 |
| Starch | 38.50 | |
| Crude Fiber | 8.02 | |
| Neutral detergent fiber | | 59.38 |
| Acid detergent fiber | | 41.08 |
| Acid detergent lignin | | 6.89 |
| Ash | 9.78 | 6.81 |
| Net energy for lactation, Mcal/kg of DM | 1.85 | 1.22 |
