# Supplementary material for: Effect of the Lactation Phases on the Amplitude of Variation in Blood Serum Steroid Hormones and Some Hematochemical Analytes in Three Dairy Cow Breeds
Source: Animals (Basel). 2024 Nov 20;14(22):3336. doi: 10.3390/ani14223336 (PMC11591377; doi:10.3390/ani14223336)
Supplement: Supplementary file 1 [file animals-14-03336-s001.zip › Table S2.pdf]

Table S2. Mean ± SD temperature and humidity recorded daily in Ragusa (https://www.wunderground.com/dashboard/pws/IRAGUSAD2), where the cows included in the present study were bred, and related Temperature Humidity Index (THI). To limit heat stress, during the hot period pasture was not available and the livestock housing was equipped with automatic system fans and freely accessible showers.

	Temperature (°C)	Humidity	THI	Lactation stage	Pregnancy stage
**February 2021**	12.9 ± 3.00	0.8 ± 0.06	55.7 ± 4.55	0 - 60 d	NP
**March 2021**	13.5 ± 1.41	0.4 ± 0.10	56.9 ± 1.62	0 - 60 d	NP
**April 2021**	16.3 ± 2.57	0.3 ± 0.06	60.1 ± 2.63	>60 - 120 d	NP
**May 2021**	20.3 ± 1.55	0.3 ± 0.06	64.3 ± 1.41	>60 - 120 d	NP
**June 2021**	25.0 ± 3.44	0.3 ± 0.07	69.3 ± 3.05	>120 - 180 d	0 - 60 d
**July 2021**	27.1 ± 1.65	0.3 ± 0.07	72.2 ± 1.40	>120 - 180 d	0 - 60 d
**August 2021**	28.1 ± 1.89	0.3 ± 0.07	73.6 ± 1.54	>180 - 240 d	>60 - 120 d
**September 2021**	25.5 ± 1.09	0.4 ± 0.07	71.3 ± 1.63	>180 - 240 d	>60 - 120 d
**October 2021**	20.3 ± 1.99	0.4 ± 0.11	65.2 ± 2.65	>240 - 300 d	>120 - 180 d
**November 2021**	18.5 ± 2.77	0.5 ± 0.13	63.4 ± 3.67	>240 - 300 d	>120 - 180 d
**December 2021**	13.1 ± 1.82	0.4 ± 0.12	56.5 ± 2.24	>300 d	>180 d
**January 2022**	11.7 ± 1.86	0.4 ± 0.11	54.8 ± 2.04	>300 d	>180 d
**February 2022**	12.9 ± 1.13	0.4 ± 0.15	56.3 ± 1.27		>180 d
